# Pharmacokinetics of abacavir and lamivudine in infants living with HIV with and without rifampicin co-treatment

**DOI:** 10.1093/jac/dkag281

**Published:** 2026-08-20

**Authors:** Laize Sílvia dos Anjos Botas Beca, Vivian Mumbiro, Mutsa Bwakura, Hilda A Mujuru, Shepherd Mudzingwa, Alfredo Tagarro, Sara Domínguez-Rodríguez, Victor Musiime, Damalie Nalwanga, W Chris Buck, Jahit Sacarlal, Chishala Chabala, Bwendo Nduna Chansa, Pablo Rojo, David M Burger, Cinta Moraleda, Tom G Jacobs, EMPIRICAL Clinical Trial Group, Alfeu Passanduca, Uneisse Cassia, Muhammad Sidat, Elias Manjate, Sónia Martins, Stella Langa, Natália Nipaco, Sara Machava, Anastância Chirindza, Luzidina Martins, Mércia Nhaca, Kusum J Nathoo, Moses Chitsamatanga, Ruth Marange, Dorothy Murungu, Natasha Namuziya, Idah Zulu, Perfect Shankalala, Mulima Mukubesa, Juliet Namwinwa, Chalwe Chibuye, Terence Chipoya, Veronica Mulenga, Bwalya Simunyola, John Tembo, Muleya Inambao, Salome Chitondo, Wyclef Mumba, Endreen Mankushe, Henry Musukwa, Davies Sondashi, Albert Kamugisha, Karen Econi, Andrew Kiggwe, Judith Beinomugisha, Sharafat Nkinzi, Lawrence Kakooza, Henriator Namisanvu, Nancy Lajara Mark, Josam Thembo Mwesige, Ivan Segawa, Joseph Ssessanga, Paul Mbavu, Bosco Kafufu, Denis Nansera, Elizabeth Najjingo, Bashira T Mbabazi, Abbas Lugemwa, Mariam Kasozi, Rogers Ankunda, Lilit Manukyan

**Affiliations:** Department of Pharmacy, Lúrio University, Nampula, Mozambique; College of Health Sciences, Clinical Research Centre, University of Zimbabwe, Harare, Zimbabwe; College of Health Sciences, Clinical Research Centre, University of Zimbabwe, Harare, Zimbabwe; College of Health Sciences, Clinical Research Centre, University of Zimbabwe, Harare, Zimbabwe; College of Health Sciences, Clinical Research Centre, University of Zimbabwe, Harare, Zimbabwe; Instituto de Investigación Sanitaria Hospital 12 de Octubre (imas12)—Fundación Biomédica del Hospital Universitario 12 de Octubre (FIB-H12O) Madrid, Spain; Department of Pediatrics, Hospital Universitario Infanta Sofia, SERMAS, Madrid, Spain; Instituto de Investigación Sanitaria Hospital 12 de Octubre (imas12)—Fundación Biomédica del Hospital Universitario 12 de Octubre (FIB-H12O) Madrid, Spain; Faculty of Medicine and Health Sciences, Makerere University, Kampala, Uganda; Faculty of Medicine and Health Sciences, Makerere University, Kampala, Uganda; David Geffen School of Medicine, University of California Los Angeles, Los Angeles, USA; School of Medicine, Universidade Eduardo Mondlane, Maputo, Mozambique; School of Medicine, Universidade Eduardo Mondlane, Maputo, Mozambique; Herpez, Lusaka, Zambia; School of Medicine, University of Zambia, Lusaka, Zambia; Arthur Davidson Children's Hospital, Ndola, Zambia; Instituto de Investigación Sanitaria Hospital 12 de Octubre (imas12)—Fundación Biomédica del Hospital Universitario 12 de Octubre (FIB-H12O) Madrid, Spain; Universidad Complutense, Madrid, Spain; Hospital Universitario 12 de Octubre, Servicio Madrileño de Salud (SERMAS), Madrid, Spain; Department of Pharmacy, Pharmacology and Toxicology, Radboudumc Institute for Medical Innovation (RIMI), Radboud University Medical Center, 864 Radboudumc, Geert Grooteplein 10, Nijmegen 6525 GA, The Netherlands; Instituto de Investigación Sanitaria Hospital 12 de Octubre (imas12)—Fundación Biomédica del Hospital Universitario 12 de Octubre (FIB-H12O) Madrid, Spain; Hospital Universitario 12 de Octubre, Servicio Madrileño de Salud (SERMAS), Madrid, Spain; Department of Pharmacy, Pharmacology and Toxicology, Radboudumc Institute for Medical Innovation (RIMI), Radboud University Medical Center, 864 Radboudumc, Geert Grooteplein 10, Nijmegen 6525 GA, The Netherlands; Department of Pharmacy, Tergooi Medical Centre, Hilversum, The Netherlands

## Abstract

**Background:**

Lamivudine and abacavir are key components of first-line paediatric antiretroviral therapy (ART). Rifampicin is part of drug-sensitive TB treatment in children. Rifampicin induces UDP-glucuronosyltransferases and renal transporters and inhibits gastrointestinal transporters involved in the pharmacokinetics of abacavir and lamivudine, but pharmacokinetic data on this potential interaction in infants are scarce.

**Objective:**

To evaluate the effect of rifampicin on the pharmacokinetics of abacavir and lamivudine in infants aged 28–365 days and weighing >3 kg.

**Methods:**

This pharmacokinetic sub-study was nested within the EMPIRICAL trial (NCT03915366). Eighteen infants from Mozambique, Uganda, Zambia and Zimbabwe received once-daily fixed-dose abacavir/lamivudine with dolutegravir according to WHO guidelines. Five infants received ART alone and 13 received ART plus rifampicin-based tuberculosis treatment. Abacavir/lamivudine dosing was unchanged during rifampicin co-treatment. Six plasma concentrations were measured over 24 h and analysed using non-compartmental pharmacokinetic analysis.

**Results:**

Median (IQR) age and weight were 6.8 (5.2–10.2) months and 6.0 (5.3–6.8) kg, respectively, and were comparable between groups. Abacavir exposure was comparable with and without rifampicin (AUC_0–24h_: 21.9 versus 21.0 h mg/L; *C*_max_: 6.2 versus 6.3 mg/L, respectively). Lamivudine exposure was also comparable (AUC_0–24h_: 12.6 versus 12.4 h mg/LL, respectively). The slightly higher *C*_max_ (2.4 versus 1.9 mg/L) in the rifampicin group is probably due to interindividual variability or increased absorption from rifampicin's inhibition of gastrointestinal drug transporters, and considered not clinically relevant.

**Conclusions:**

Rifampicin co-treatment had minimal impact on abacavir and lamivudine pharmacokinetics in infants. Dose adjustment of abacavir/lamivudine is not required during rifampicin-containing tuberculosis treatment.

## Introduction

TB and HIV remain significant global health issues, with ∼80% of cases occurring in low- and middle-income countries, affecting all age groups.^1^ In 2023, it was estimated that TB caused 1.25 million deaths, including 161 000 among people living with HIV.^1^ Furthermore, 700 000 children and young adolescents (0–14 years) were diagnosed with TB.^1^ Children living with HIV are at higher risk of developing TB disease and are more likely to experience a more severe course of both infections.^2^ Concomitant administration of multiple drugs for treatment of both infections is inevitable in these situations. However, this may result in pharmacokinetic drug–drug interactions (DDIs) that may compromise the treatment’s efficacy and safety.^3^

According to WHO guidelines, first-line antiretroviral therapy (ART) for infants living with HIV with TB co-infection consists of abacavir/lamivudine and dolutegravir alongside with rifampicin-based TB treatment.^4^ The co-administration with rifampicin, a potent inducer of drug-metabolizing enzymes, necessitates an increase in the dolutegravir dose to twice daily, whereas the doses of abacavir and lamivudine remain unchanged.^4,5^

Rifampicin is a potent inducer of cytochrome P450 enzymes, UDP-glucuronosyltransferases (UGT), and renal drug transporters and inhibits various gastrointestinal drug transporters.^6^ Abacavir is primarily metabolized by alcohol dehydrogenase and UGT2B7; owing to induction of the latter, rifampicin co-administration may lead to reduced systemic abacavir concentrations.^7,8^ Lamivudine is renally excreted via passive glomerular filtration and active tubular secretion, mediated by Organic Cationic Transporter-2 (OCT-2) and Multidrug and Toxin Extrusion Transporters 1 and 2 (MATE1 and MATE2).^9^ Rifampicin is known to induce various renal transporters and may affect elimination of lamivudine.^10^ Despite this, and surprisingly, the effect of rifampicin on lamivudine pharmacokinetics remains unstudied to our knowledge. Therefore, this sub-study aimed to assess the effect of rifampicin co-administration on the pharmacokinetics of abacavir and lamivudine in infants living with HIV receiving an ART regimen consisting of abacavir/lamivudine with dolutegravir.

## Methods

### Study population and design

This was an open-label, multicentre pharmacokinetic (PK) sub-study conducted within the EMPIRICAL randomized controlled trial (NCT03915366).^11^ The EMPIRICAL trial enrolled hospitalized infants aged 28 to 365 days with severe HIV-associated pneumonia to assess whether empiric treatment for cytomegalovirus and tuberculosis improves survival using a 2 × 2 factorial design, enabling the comparison of ART regimen PK with and without rifampicin. This sub-study included infants from clinical centres in Mozambique, Uganda, Zambia and Zimbabwe.

### Procedures

All infants received a fixed-dose dispersible combination (FDC) of abacavir/lamivudine along with dolutegravir dispersible tablets, following WHO dosing guidelines.^4^ The infants either received abacavir/lamivudine plus dolutegravir once daily without TB treatment or abacavir/lamivudine once daily plus dolutegravir twice daily with TB treatment; paediatric FDC isoniazid/rifampicin/pyrazinamide. Adherence to ART during the 3 days preceding the PK visit was reported by caregivers. We used residual samples from a previous sub-study that were collected pre-dose and 2, 4, 6, 8 and 12/24 hours after abacavir/lamivudine dose administration after being on rifampicin for at least 30 days.^5^ The exact collection times were recalibrated relative to the abacavir/lamivudine dose administration for pharmacokinetic evaluation. All concentrations were quantified Radboudumc (Nijmegen, The Netherlands) using a validated LC-MS/MS assay. The lower and upper limit of quantification were 0.015 and 5.0 mg/L for both analytes. The EMPIRICAL trial protocol, including the pharmacokinetic sub-studies, was approved by local ethics committees and national ethical and regulatory authorities. Caretakers provided written informed consent for participation in the PK sub-study and verbal consent on the PK sampling day, with study documents translated into local languages.

### Data analysis

Participant characteristics (age, sex, weight, eGFR, nutritional status and dosage) were collected. Pharmacokinetic parameters were calculated via non-compartmental analysis using WinNonlin v.8.3, including the 24-hour area under the plasma concentration–time curve (AUC_0–24h_), maximum concentration (*C*_max_), and time to *C*_max_ (*T*_max_). For each parameter, geometric means (GM) and intersubject coefficients of variation (CV%) were calculated. Abacavir and lamivudine AUC_0–24h_ were compared to the reference ranges used for paediatric approvals of dispersible abacavir/lamivudine/dolutegravir (Triumeq^®^) tablets: abacavir AUC_0–24h_ 6.3–50.4 h*mg/L and lamivudine AUC_0–24h_ 6.3–26.5 h*mg/L.^12,13^ These ranges represent the lower and upper bound of the 90% confidence intervals for predicted exposures with once-daily administration in children living with HIV.

## Results

### Demographics

The sub-study group included 18 infants (six females and 12 males) whose demographics are detailed in Table 1. Of all included infants, 13 received concomitant rifampicin and five did not receive rifampicin co-treatment.

**Table 1.** Infant characteristics and pharmacokinetic results

	Control arm (*n* = 5)	Rifampicin arm (*n* = 13)
Characteristics		
Sex: % female	40%	31%
Age, months; median (IQR)	7.1 (6.2 to 11.1)	6.3 (3.2 to 11.9)
Weight, kg; median (IQR)	6.0 (5.7 to 8.2)	5.9 (3.8 to 8.0)
Infants in WHO weight band 3 < 6 kg	2 (40%)	7 (54%)
Infants in WHO weight band 6 < 10 kg	3 (60%)	6 (46%)
Length, cm; median (IQR)	64.0 (60.0 to70.0)	59 (53.0 to 68.0)
Baseline HIV viral load, log_10_ copies/mL; median (IQR)	5.00 (1.60 to 7.00)	5.90 (2.09 to 7.21)
Pharmacokinetic results		
Abacavir AUC_0–24h_ (h mg/L)	21.0 (19)	21.9 (74)
Abacavir *C*_max_ (mg/L)	6.3 (55)	6.2 (10)
Abacavir *T*_max_ (h)	1.96 (18)	2.00 (5)
Lamivudine AUC_0–24h_ (h mg/L)	12.4 (37)	12.6 (49)
Lamivudine *C*_max_ (mg/L)	1.9 (21)	2.4 (52)
Lamivudine *T*_max_ (h)	3.02 (39)	2.5 (32)

All demographic data are expressed as median (IQR) and all pharmacokinetic parameters are expressed as geometric mean (%coefficient of variation) unless stated differently.

### Pharmacokinetic analysis

**Figure 1.**
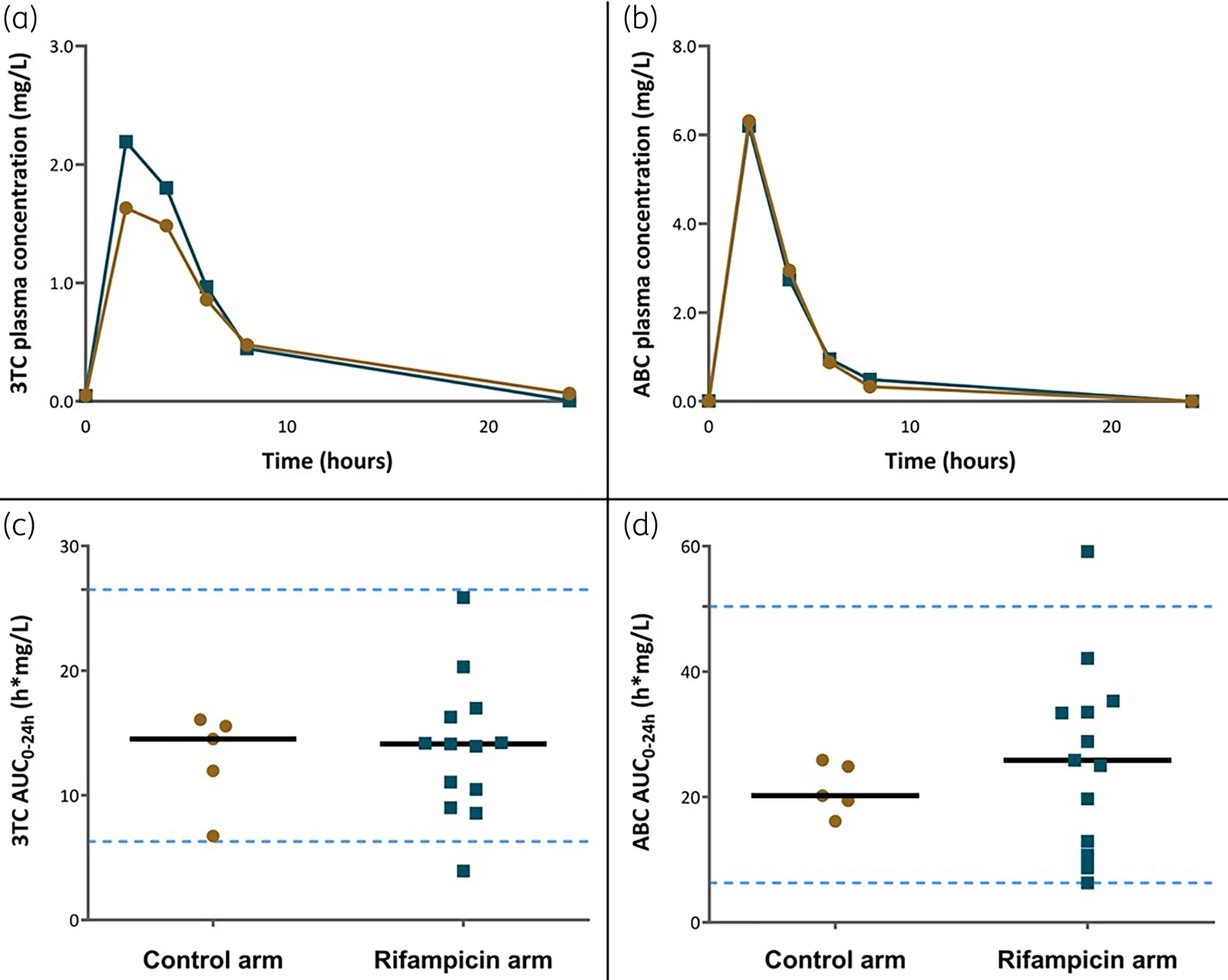
Geometric mean plasma concentration–time curves for children receiving lamivudine (3TC) (a) and abacavir (ABC) (b), and individual AUC_0–24h_ values for lamivudine (c) and abacavir (d). The brown dots represent infants receiving lamivudine/abacavir without rifampicin and the blue squares represent infants with rifampicin.

The co-administration of rifampicin did not result in relevant changes to the pharmacokinetics of abacavir or lamivudine (see Table 1 and Figure 1). For abacavir, the GM AUC_0–24h_ and *C*_max_ were 21.0 h mg/L and 6.3 mg/L without rifampicin versus 21.9 h mg/L and 6.2 mg/L with rifampicin, respectively. For lamivudine, the GM AUC_0–24h_ and *C*_max_ were 12.4 h mg/L and 1.9 mg/L without rifampicin compared with 12.6 h mg/L and 2.4 mg/L with rifampicin, respectively. Individual abacavir and lamivudine AUC_0–24h_ values by weight band are included in Figure S1 (available as Supplementary data at *JAC* Online).

One infant receiving rifampicin had an abacavir AUC_0–24h_ above the reference range (59.2 h mg/L), and one had a lamivudine AUC_0–24h_ below the reference range (3.93 h mg/L). All other infants in both groups had AUC_0–24h_ values within the reference ranges for both abacavir and lamivudine.

## Discussion and conclusion

This pharmacokinetic sub-study evaluated the effect of rifampicin co-treatment on abacavir and lamivudine pharmacokinetics in infants living with HIV aged 28 to 365 days. The findings demonstrate that concomitant administration of rifampicin resulted in minimal alterations in the pharmacokinetics of both drugs. The GM AUC_0–24h_ and *C*_max_ values of abacavir and lamivudine were comparable between infants receiving and not receiving rifampicin, suggesting that no relevant DDI occurs under these conditions.

The abacavir and lamivudine exposures, both with and without rifampicin, in this sub-study are comparable to those reported in previous studies.^12,13^ In the registrational study of paediatric abacavir/lamivudine/dolutegravir FDC tablets the GM AUC_0–24h_ and *C*_max_ values were 17.7 h mg/L and 7.3 mg/L for abacavir and 10.7 h mg/L and 2.29 mg/L for lamivudine, respectively, in children.^12^ The consistency of these findings with our results confirms that abacavir/lamivudine exposures are appropriate regardless of rifampicin use.

The absence of a significant impact of rifampicin on abacavir exposure was unexpected, given that rifampicin is a known inducer of UGT enzymes, one of the primary metabolic pathway for abacavir.^6^ However, alcohol dehydrogenase also contributes to metabolism of abacavir and is not induced by rifampicin. A previous study in children showed a 36% reduction in abacavir AUC among children receiving rifampicin with super-boosted lopinavir/ritonavir compared with children receiving a regular dose of lopinavir/ritonavir without rifampicin.^7^ The authors indicated that it remained unclear whether the decreased abacavir exposure was due to the extra ritonavir (also a UGT2B7 inducer) added to the super-boosted lopinavir/ritonavir or the rifampicin co-treatment.^7^ Our findings suggest that rifampicin alone has minimal effect on abacavir exposure. This may be partially explained by the inclusion of younger children in our sub-study as hepatic UGT2B7 activity is lower in early infancy and therefore may be less prone to induction by rifampicin.^14^ Interestingly, a descriptive weight-stratified analysis of our data suggested lower abacavir exposure in infants weighing 6–<10 kg compared with those weighing 3–<6 kg, whereas a similar pattern was not observed for lamivudine. However, no association between age and abacavir exposure was observed. These findings should be interpreted in context of the small sample size.

Similarly, lamivudine pharmacokinetics were not meaningfully altered by rifampicin co-treatment, suggesting that rifampicin does not meaningfully induce renal transporters involved in lamivudine elimination, namely OCT-2, MATE1 and MATE2. The slightly higher *C*_max_ observed in the rifampicin group (2.4 versus 1.9 mg/L) is likely attributable to interindividual variability or inhibition of gastrointestinal drug transporters by rifampicin.^6^ This difference in *C*_max_ is not considered clinically relevant, especially as it is accompanied by a slightly faster elimination, which results in comparable overall AUC.

The novel paediatric once-daily FDC of abacavir/lamivudine/dolutegravir is increasingly used in low- and middle-income countries under WHO guidelines.^4^ Consequently, the availability of stand-alone paediatric dolutegravir tablets may decline in the future. This poses a challenge as co-administration with rifampicin-based tuberculosis treatment requires dolutegravir to be given twice daily rather than once daily,^5^ while the abacavir/lamivudine backbone should remain once daily. Our findings support the current WHO recommendation that abacavir and lamivudine doses do not require adjustment in presence of rifampicin. Future studies evaluating safety of twice-daily abacavir/lamivudine dosing are needed to enable twice-daily administration of the FDC when required. Of note, initial pharmacokinetic modelling studies suggest that once-daily dolutegravir dosing may be sufficient in presence of rifampicin, but clinical studies confirming adequacy of this approach are still ongoing.^5^

Our sub-study has several limitations including abacavir/lamivudine not being provided as an investigational product of the EMPIRICAL trial. Therefore, infants may have received different brands of the abacavir/lamivudine dispersible tablets, which were not recorded. In addition, only abacavir/lamivudine plasma concentrations were measured while intracellular triphosphate concentration are more closely related to antiviral efficacy. However, plasma pharmacokinetics are the standard endpoint in DDI studies and are appropriate for assessing the magnitude of a DDI. Finally, the smaller sample size in the control arm relative to the rifampicin arm may have affected the robustness of between-group PK comparisons. Despite this, the pharmacokinetic parameters found in our sub-study were comparable to those reported in previous studies evaluating abacavir and lamivudine pharmacokinetics in children not receiving rifampicin.^12,13^

Our data demonstrate that rifampicin co-treatment had minimal impact on the pharmacokinetics of abacavir or lamivudine in infants living with HIV. These findings confirm that the standard WHO-recommended doses of abacavir and lamivudine can be maintained during rifampicin-based tuberculosis co-treatment. Future studies are needed to study whether dolutegravir could be dosed once daily or whether abacavir/lamivudine could be dosed twice daily to facilitate dosing of dolutegravir/abacavir/lamivudine fixed-dose dispersible tablets in presence of rifampicin.

## Supplementary Material

dkag281_Supplementary_Data

## Data Availability

Data is available upon reasonable request from the corresponding author.

## Supplementary data

Figure S1 is available as Supplementary data at *JAC* Online.
