# Supplementary material for: Pharmacokinetics of abacavir and lamivudine in infants living with HIV with and without rifampicin co-treatment
Source: J Antimicrob Chemother. 2026 Aug 20;81(9):dkag281. doi: 10.1093/jac/dkag281 (PMC13492079; doi:10.1093/jac/dkag281)
Supplement: Supplementary file 1 [file dkag281_supplementary_data.docx]

Supplementary material

**Supplementary Figure 1. Exposure to abacavir and lamivudine stratified by weight band.** Individual AUC_0-24h_ values for abacavir and lamivudine are shown for each infant. The y-axis represents individual AUC_0-24h_ values , and the x-axis shows the two weight bands (3–<6 kg and 6–<10 kg). Data are presented irrespective of rifampicin co-administration.
